# Examining the perceived versus the actual knowledge about forensic odontology: A cross‐sectional survey among dentists

**DOI:** 10.1002/cre2.148

**Published:** 2018-12-13

**Authors:** Adel F. Almutairi, Bayan A. Alkhtheri, Hattan N. Aleidan, Asma A. Alhabib, Eid A. Alotaibi, Mahmoud Salam

**Affiliations:** ^1^ Science and Technology Unit ‐ Ministry of National Guard ‐ Health Affairs King Abdullah International Medical Research Center/King Saud bin Abdulaziz University for Health Sciences Riyadh Saudi Arabia; ^2^ Riyadh Elm University Riyadh Saudi Arabia; ^3^ College of dentistry—Ministry of National Guard—Health Affairs King Abdullah International Medical Research Center/King Saud bin Abdulaziz University for Health Sciences Saudi Arabia; ^4^ College of Dentistry Al Qassim University Al Qassim Saudi Arabia

**Keywords:** dentists, forensics, knowledge, perceived, Saudi Arabia, willingness

## Abstract

Dentists should have the basic essential skills and knowledge about forensic odontology, to better collaborate with law enforcement and investigations. The objective of this survey was to assess the perceived and actual knowledge toward forensic odontology among dentists and to question their willingness to attend training courses on this specialty. A cross‐sectional survey based on a self‐administered questionnaire was conducted in various districts of Saudi Arabia. Four hundred dentists responded to a questionnaire that tested their actual knowledge of forensic odontology based on answering 15 statements using the alternatives correct, incorrect, do not know. The perceived knowledge was registered as strongly agree, agree, neutral, disagree, and strongly disagree, then assigned scores respectively from four to zero. A willingness to attend a training course in the future was recorded by (yes/no). Scores were summated then subjected to descriptive statistics and regression analyses. Responses were received from 360 study participants (89% response rate). The percentage of correct answers, that is, the actual knowledge, was 67.9 (standard deviation [*SD*] ± 18.4). About two thirds of the responders (*n* = 251, 69.7%) indicated a willingness to attend a forensic odontology course in the future. Differences in both actual and perceived knowledge were identified on the basis of gender, work experience, education level, attended a course in forensic odontology, and having previously provided a past bite‐mark examination. The perceived knowledge on forensic odontology among dentists was moderate to low. The gap between perceived and actual knowledge signifies low self‐confidence. Dentists with higher education levels and experience tend to have better knowledge.

**List of abbreviations**
 percentage mean score (PMS)
 standard deviation (± *SD*)

1

**Key Points**

- The concept of comparing the perceived versus the actual knowledge about forensic odontology has been seldom studied.
- A gap between the perceived and actual knowledge can be either due to misconception or a low degree of self‐confidence; in this setting, it indicated poor self‐confidence.
- The level of knowledge about forensic odontology among dentists in Saudi Arabia was moderate to low.
- Optimal desired knowledge is an ultimate goal that will not be achieved unless researchers measure the willingness of dentists to attain specialized training programs.
- Special consideration should be made for less educated dentists (bachelor degree), less experienced (<2 years) dentists, and those working in private clinics as they tend to have poorer knowledge.

## 2 INTRODUCTION

Forensic odontology is a branch of dentistry that applies dental knowledge to criminal and civil law enforcement through the examination of dental evidence (Gambhir, Singh, Talwar, Gambhir, & Munjal, 2016). Forensic odontologists play a significant role through the examination of anatomical structures, dental appliances, and dental restorations (Gambhir et al., 2016; Srinivasa, Sujatha, Sivakumar, & Muruganandhan, 2012). Because the integration of forensic odontology into forensic medicine in the past few decades, dentists have assisted in the identification of human remains, assessment of bite‐mark injuries, estimation of age, and investigation of suspected social abuse (children, spouses, or elders; Jyothi, Bhanu, Bhaskar, Kumar, & Sujith, 2017).

It has been recommended that dental practitioners should have the necessary skills and knowledge about forensic odontology, such as handling dental records, bite‐mark examination, teeth–pulp DNA analysis, radiographs, teeth morphology, and anatomy (Stavrianos, Kokkas, Andrepuolos, & Eliades, 2010). In cases where a fingerprint record or physical identification is missing, the responsibility shifts to dental practitioners to contribute in crime investigations (Singh, Gowhar, Ain, & Sultan, 2014). One approach is to perform lip print analysis, which has a unique wrinkle pattern and characteristics that can lead to the identification of an individual. This science is called cheiloscopy, a forensic investigation technique that deals with identification of people based on lips traces (Dineshshankar, Ganapathi, Kumar, Aravindhan, & Maheswaran, 2013). Forensic odontologists also assess dental records to investigate the identity of unknown person's identity. For example, they compare the dental data of a missing individual (antemortem) with that of a deceased person (postmortem; Interpol, n.d.).

Despite the significance of this field in criminal justice, the literature indicates that forensic odontology is underdeveloped in many countries and steps are needed to improve the use of this science (Kumar & Dagli, 2014). For instance, in the United States, four organizations are dedicated to forensic odontology: the Bureau of Legal Dentistry, the American Board of Forensic Odontology, the American Society of Forensic Odontology, and the International Organization for Forensic Odonto‐Stomatology (Colvard et al., 2016). Other countries, such as the United Kingdom and Australia, have realized its significance and established the British Association for Forensic Odontology and the Australian Society of Forensic Odontology (Colvard et al., 2016). Dental practitioners' knowledge needs to be evaluated and then improved, if necessary, by mandating courses on forensic odontology and engagement in investigating cases to improve their hands‐on expertise. Skilled dentists with expertise in forensic odontology are in continuous demand (Singh et al., 2014). The aim of this study was to assess the level of knowledge toward forensic odontology among Saudi dentists and to question their willingness to attend training courses related to this dental speciality.

## 3 MATERIALS AND METHODS

A cross‐sectional study was conducted between July and August 2017, using a self‐administrated anonymous survey distributed by convenience among dentists working in various districts of Saudi Arabia. Ethical approval to conduct this study was issued by the Institutional Review Board (RSS 17/010) at King Abdullah International Medical Research Center, Ministry of National Guard Health Affairs. Students of dentistry were excluded. The participation in the survey was voluntary, verbal consent was obtained, and the confidentiality of the participants was maintained by the data collectors.

The participants were invited to provide information about their gender, age, years of clinical experience, level of education, work sector (private or governmental), and whether they had attended forensic odontology course in the past and/or whether they had provided any bite‐mark consultations. Their actual knowledge on forensic odontology was appraised by evaluating their responses for 15 statements, sourced from published literature (Ali, Sardar, Nasir, & Wakar, 2016; Bhakhri, Kaur, Singh, Puri, & Puri, 2017; Sahni, Rehani, Mathias, Kardam, & Nagpal, 2016), with three alternative responses, that is, correct, wrong, or do not know. One additional question was their perceived level of knowledge, with answers rated on a 5‐point Likert scale (*strongly disagree*, *disagree*, *neutral*, *agree*, and *strongly agree*). A final question was about the willingness to attend a forensic odontology course (yes or no). The summated score of the 15 actual knowledge statements was obtained for each study participant (correct responses scored 1, and wrong and do not know responses scored 0), before the percentage mean scores were calculated.

Data were entered and analyzed by using the Statistical Package for Social Sciences (SPSS) statistical software version 25 (International Business Machines [IBM], SPSS Inc, NY). Descriptive statistics (frequencies and percentages) were used to present categorical sample characteristics and responses, whereas the arithmetic mean and standard deviation (*SD*) were used to present the outcomes. Bivariate analysis was conducted using an independent Student *t* test (*t*), one‐way ANOVA (*F*), and Pearson's chi‐square (χ^2^) to present the outcomes across various exposures. Two linear regression analysis models were constructed to identify factors associated with higher perceived and actual knowledge. One logistic regression analysis model was constructed to identify exposures associated with the dentists' willingness to attend training courses in forensic odontology. Statistical significance was set at P < 0.05.

## 4 RESULTS

A total of 360 dentists participated in this study (response rate, 89%). Almost equal gender distribution was observed. Those with age <30 years were 205 (56.9%), whereas those ≥30 were 155 (43.1%), mean ± *SD* of age (30.5 ± 6.8 years). Level of education ranged between bachelor, 259 (71.9%), master's degree, 84 (23.4%), and Ph.D., 17 (4.7%). Those with experience <2 years were 88 (24.4%), followed by 2–5 years, 128 (35.6%), then >5 years, 144 (40.0%). Dentists working in the government sector were 142 (39.4%), whereas 218 (60.6%) worked in the private sector. Majority of dentists in this study have not provided any dental consultation for a bite victim in the past, 328 (91.1%), and only few, 95 (26.4%), ever attended a course in forensic odontology.

**Figure 1:**
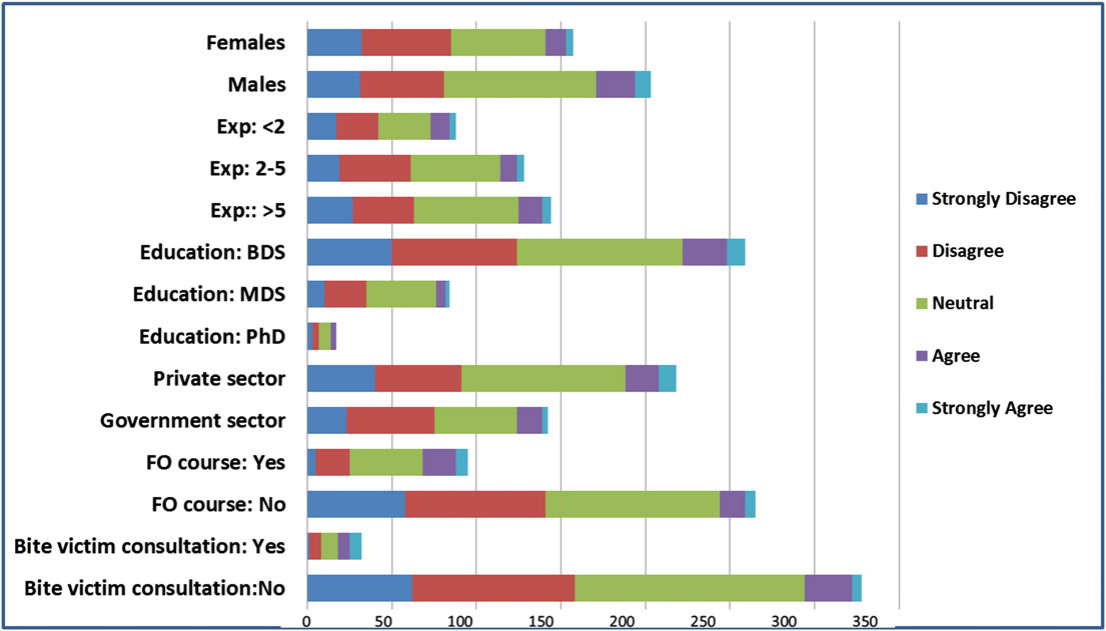
Perceived knowledge of forensic odontology between the subgroups

The proportions of correct responses for the individual statements varied (Table 1), with an overall correct response rate of 67.9 (*SD* ± 18.4). The highest proportion of correct responses was reported in Questions 2 (320, 88.9%), 6 (308, 85.6%), and 14 (304, 84.4%). This reflected a high level of knowledge on most of the important aspects of forensic dentistry, which are investigations of physical violence, estimation of individuals' age, and bite‐mark analyses. However, the lowest proportion of correct responses was reported in the Questions 8 (72, 20.0%), 4 (74, 20.6%), and 9 (81, 22.5%). This showed that dentists had knowledge deficiencies that were mainly related to the significance of forensic odontology on other aspects such gender discrimination and child‐neglect investigations. Approximately 22% of the participants demonstrated good actual knowledge (correct scores for ≥80% of the questions), 53% demonstrated average knowledge, and 25% demonstrated poor knowledge (correct scores on ≤60% of the questions). The perceived knowledge of forensic odontology was reported as being in *strongly agreement* (13, 3.6%), *agreement* (35, 9.7%), *neutrality* (146, 40.6%), *disagreement* (103, 28.6%), and *strongly disagreement* (63, 17.5%). The frequency distributions varied among the study participants as a function of several characteristics (Figure 1). The actual knowledge (PMS ± *SD* = 67.9 ± 18.4), perceived knowledge (*MS* ± *SD* = 1.5 ± 1.0), and study participants' willingness to partake in a course in forensic odontology (251, 69.7%) are shown in Table 2. Significant differences between different subgroups were noted. Dentists with experience above 5 years (PMS = 69.7 ± 17.3), those who are Ph.D. educated (PMS = 75.3 ± 11.5), those working in government sector (PMS = 70.3 ± 15.4), and those who attended previous forensic odontology in the past (PMS = 71.4 ± 15.9) had significantly higher actual knowledge PMS compared with their comparable subgroups (P = 0.040, P = 0.021, P = 0.042, and P = 0.020, respectively). Dentists who previously attended a course (*MS* = 2.0 ± 0.9) and who provided a past bite‐mark consultation in the past (*MS* = 2.4 ± 1.1) had significantly higher perceived knowledge than their comparable subgroups, P < 0.001 each.

**Table 1:** Responses to the forensic odontology knowledge items

	Item	Correct	Wrong	I don't know
		*n* (%)	*n* (%)	*n* (%)
1	Forensic odontology aids in the physical violence identification.	289 (80.3)	33 (9.2)	38 (10.5)
2	Forensic odontology aids in the estimation of an individual's age.	320 (88.9)	17 (4.7)	23 (6.4)
3	Forensic odontology aids in gender identification of victims.	83 (23.1)	192 (53.3)	85 (23.6)
4	Forensic odontology can help confirm child neglect.	74 (20.6)	209 (58.1)	77 (21.3)
5	Forensic odontology can help in the investigating sexual abuse.	131 (36.4)	158 (43.9)	71 (19.7)
6	Analysis of bite‐mark patterns aids in identifying criminals.	308 (85.6)	17 (4.7)	35 (9.7)
7	Dental patterns are unique identifiers.	278 (77.2)	30 (8.3)	25 (14.5)
8	An individual has a unique lip print.	72 (20.0)	163 (45.3)	125 (34.7)
9	An individual has a unique jaw structure.	81 (22.5)	195 (54.2)	84 (23.3)
10	DNA can be extracted from the teeth of a deceased person.	231 (64.2)	40 (11.1)	89 (24.7)
11	Palatal rugae can be used as a marker in forensic identification.	187 (51.9)	47 (13.1)	126 (35.0)
12	Practicing forensic odontology needs permit or certification.	295 (81.9)	65 (18.1)	0 (0.0%)
13	An individual dental age can be estimated by radiography.	299 (83.1)	49 (13.6)	12 (3.3)
14	An individual dental age can be estimated by the eruption status.	304 (84.4)	39 (10.8)	17 (4.8)
15	The most accurate and sensitive method to identify an individual is as follows:			*n* (%)
		‐Visual identification		20 (5.6%)
		‐Finger prints		51 (14.2%)
		‐Physical anthropological exam of bone and teeth		24 (6.7%)
		‐DNA comparison		365 (73.6%)*

*Note*. *n*: frequency; %: percentage.

* It signifies the correct answer.

**Table 2:** The level of knowledge toward forensic odontology among dentists and their willingness to attend a course across sample characteristics

Exposures	Actual knowledge	Perceived knowledge	Willingness to attend a course
	% mean score	Likert mean score	*n* (%)
	67.9 ± 18.4	1.5 ± 1.0	251 (69.7%)
Gender			
Females	67.6 ± 18.9	1.4 ± 1.0	117 (74.5%)
Males	68.1 ± 18.0	1.7 ± 1.0	134 (66.7%)
	*t* = −0.297, P = 0.766	*t* = −2.537, P = 0.012*	χ^2^ = 2.596, P = 0.107
Experience (years)			
<2	63.6 ± 18.2	1.5 ± 1.1	64 (73.6%)
2–5	68.8 ± 19.4	1.5 ± 0.9	89 (69.5%)
>5	69.7 ± 17.3	1.5 ± 1.0	98 (68.5%)
	*F* = 3.241, *df* = 2, P = 0.040*	*F* = 0.031, *df* = 2, P = 0.970	χ^2^ = 0.686, *df* = 2, P = 0.710
Level of education			
BDS	66.3 ± 19.3	1.5 ± 1.0	183 (71.2%)
MDS	71.3 ± 16.0	1.6 ± 0.9	57 (67.9%)
Ph.D.	75.3 ± 11.5	1.6 ± 1.0	11 (64.7%)
	*F* = 3.909, *df* = 2, P = 0.021*	*F* = 0.178, *df* = 2, P = 0.837	χ^2^ = 0.717, *df* = 2, P = 0.745
Work sector			
Private	66.3 ± 20.0	1.6 ± 1.0	148 (68.2%)
Government	70.3 ± 15.4	1.5 ± 0.9	103 (73.0%)
	*t* = −2.038, P = 0.042*	*t* = 1.170, P = 0.243	χ^2^ = 0.958, P = 0.328
Attended forensic odontology course			
No	66.6 ± 19.1	1.4 ± 0.9	182 (68.9%)
Yes	71.4 ± 15.9	2.0 ± 0.9	69 (73.4%)
	*t* = −2.348, P = 0.020*	*t* = −6.018, P < 0.001*	χ^2^ = 0.659, P = 0.417
Provided consultation on a bite victim in the past			
No	68.3 ± 18.0	1.5 ± 0.9	227 (69.4%)
Yes	64.1 ± 20.3	2.4 ± 1.1	24 (77.4%)
	*t* = 1.216, P = 0.225	*t* = −4.549, P < 0.001*	χ^2^ = 0.865, P = 0.352

*Note*. ANOVA: analysis of variance; BDS: Bachelor in Dental Sciences; *df*: degree of freedom; *F*: one‐way ANOVA; MDS: Masters in Dental Sciences; χ^2^: Pearson's chi‐square, %: percentage; *SD*: standard deviation; *t*: Student's *t* test.

* Statistically significant at P < 0.05.

Two linear and one logistic regression models were constructed to identify factors significantly associated with higher perceived and actual knowledge, as well as the dentists' willingness to attend a forensic odontology course, Table 3. Previously attending a course or providing a consultation on a bite mark were both significantly associated factors with higher perceived knowledge (adjusted P < 0.001 and adjusted P = 0.001, respectively). Previously attending a course was a significantly associated factor with higher actual knowledge, adjusted P = 0.015. Younger age among dentists was a significantly associated factor (adjusted odds ratio = 0.91; 95% CI [0.82–0.99]) with willingness to attend future forensic odontology courses compared with older age dentists, adjusted P = 0.036 (Table 3).

**Table 3:** Significant factors associated with the study outcomes

Exposures	Perceived knowledge	Actual knowledge	Willingness to attend a forensic odontology course
	*β* (t)	*β* (t)	*β*; adjusted OR (95% OR)
	Adjusted *p*	Adjusted *p*	Adjusted *p*
Constant	10.69 (0.92)	60.61 (6.73)	3.61; 37.0
	P = 0.359	P < 0.001*	P = 0.002*
Age (years)	0.73 (1.53)	0.283 (0.767)	−0.09; 0.91 (0.82–0.99)
	P = 0.125	P = 0.444	P = 0.036*
Gender			
Female^a^	4.23 (1.64)	0.035 (0.02)	−0.32; 0.73 (0.45–1.19)
Male^b^	P = 0.102	P = 0.986	P = 0.204
Experience (years)	−0.709 (−1.44)	−0.100 (−0.26)	0.08; 1.08 (0.98–1.20)
	P = 0.150	P = 0.793	P = 0.110
Level of education			
Less educated^a^	0.51 (0.14)	1.99 (0.69)	0.10; 1.11 (0.56–2.20)
More educated^b^	P = 0.890	P = 0.488	P = 0.767
Work sector			
Government^a^	3.82 (1.49)	−3.25 (−1.63)	−0.23; 0.79 (0.49–1.29)
Private^b^	P = 0.137	P = 0.103	P = 0.351
Attended forensic odontology course			
Not attended^a^	14.29 (4.81)	5.64 (2.45)	0.173; 1.19 (0.68–2.08)
Attended^b^	P < 0.001*	P = 0.015*	P = 0.545
Provided consultation in the past			
No^a^	15.49 (3.37)	−6.62 (−1.86)	0.43; 1.53 (0.61–3.87)
Yes^b^	P = 0.001*	P = 0.064	P = 0.368

*Note*. *β* = coefficient of determination; CI: confidence interval; OR: odds ratio; *t*: Student's *t* test.

* Statistically significant at P < 0.05.

a Reference group.

b Compared group.

## 5 DISCUSSION AND RECOMMENDATIONS

An important finding in this study is that 24.4% of the study participants demonstrated poor and 53.1%, an average knowledge of forensic odontology. Generally, the level of actual knowledge among dentists was moderately low, whereas their perceived knowledge was considered lower. A gap between the perceived and actual knowledge can be either due to misconception or a low degree of self‐confidence. The knowledge gap of dentists in this setting was wide.

Forensic odontology is based on guidelines and protocols to assist dentists in assessment and decision‐making (Vermylen, 2006). Accordingly, education and training are offered to reach the optimal desired knowledge. Achieving an optimal knowledge about forensic odontology will not be predicted unless researchers measure their willingness to attain specialized training programs. In this study, dentists who previously attended educational offerings on forensic odontology had significantly higher levels of actual and perceived knowledge, which is a logic finding. However, the focus should be on the alarming finding that the majority of dentists in this study (73.6%) have not received such courses. The optimal knowledge should be at higher levels, as crime investigators and emergency health‐care practitioners often rely on dentists' expertise to evaluate and analyze potentially incriminating dental evidence.

Previous studies have advised dentists to be extra vigilant and suspicious when dealing with victims of abuse, as domestic violence is often underrecognized or underreported (Lincoln & Lincoln, 2010). In this study, the majority of dentists agreed that forensic odontology aids in identifying physical abuse. For instance, intimate partner violence that might involve bite marks often requires a professional dental consultation, yet, in this study, only 36.4% acknowledged this fact (Sawyer, Coles, Williams, & Williams, 2016). Thus, dentists are required to possess a critical eye and expert skills when a forensic consultation is sought by legal authorities (Ahmed & Naidoo, 2017). A survey among forensic dentists showed that the majority (91%) of respondents believed that human dentition is distinctive and that 78% of bite marks on skin can be analyzed. Active involvement of dentists in protection teams of abuse or neglect victims is always beneficial and can lead to early intervention (Sujatha, Sivakumar, & Saraswathi, 2010). In this study, only 20.6% confirmed that forensic odontology can help confirm child neglect. In one of the gulf countries, a survey was conducted among dentists to recognize child abuse as well as the factors that may prevent them from reporting. Although 25% of dentists reported at least one encounter with a suspected child‐abuse case, only 32% of them reported their finding. This study also recommended specialized training to enhance the dentists' capability in diagnosing and reporting suspected child‐abuse cases (Al‐Amad, Awad, Al‐Farsi, & Elkhaled, 2016). A systematic review paper revealed that the majority of dentists had insufficient knowledge on detecting domestic violence cases, which was received in their undergraduate courses (Rodrigues, Lima, Nagata, et al., 2016). Another study emphasized on the importance of providing forensic education as part of the dentistry academic curricula, followed by proper training so that dentists become competent in the detection and management of domestic violence victims (Sawyer et al., 2016). In this study, the knowledge of dentists was moderately low, as poorer knowledge was found to be associated with less educated dentists. However, dentists who previously provided a consultation on a bite‐mark victim thought they had better knowledge about forensics, yet their actual knowledge was less than those who never provided such consultation.

Forensic odontology is indeed recognized to be one the most consistent scientific methods in disasters (De, 2009). For instance, teeth are well‐known to be resilient to various types of circumstances such as chemical attacks, burning, burial, submersion, and traumas, to certain degrees that human identification can be still possible (Pretty, 2007). During natural disasters, wars, or annual pilgrimages, forensic odontologists are capable of playing active roles within a diverse disaster management team including the police, army, and national guards. In cases where the body or remains of a person remain unidentified, a certain degree of psychological trauma will affect both family members and friends, and by rapidly evaluating reliable dental evidence, dentists are capable of confirming a positive human identification (Funaro, 2006). Although no two individual rugae patterns are alike in their configuration, only 51.9% of dentists in this study acknowledged this fact (Poojya, Shruthi, Rajashekar, & Kaimal, 2015). Majority of the dentists in this study admitted that DNA is indeed the most accurate method for identification followed by dental pattern analyses. Therefore, a team of well‐educated and trained forensic odontologists shall be readily accessible at all times.

The need for well‐established dental records is immense once a person goes missing for a long time. For instance, more than 84,000 cases of missing individuals have been reported in 2013 in the United States, of which at least 8,000 unidentified persons were enlisted in the National Crime Information Center database (Riley, 2014). A high level of cooperation has been requested by law enforcement from dentists to match the lines of unresolved cold cases. Getting a forensic dental consultation tends to be the last conventional resort in postmortem identification (Cardoza & Wood, 2015).

The empirical role of forensic odontologists in disaster management and victim identification is fulfilled by maintaining well‐structured, comprehensive, and accurate dental records for both teaching and research purposes, as well as for legal matters (Dutta, Singh, Passi, Varghese, & Sharma, 2016). Another study strongly recommended mandatory quality dental records that are both efficiently stored and easily accessible, especially in high‐risk groups such as the military populations (Guimarães et al., n.d.). The knowledge related to maintaining dental records for forensic and medicolegal purposes could be insufficient indicating a need for both proper education and training and the need for further training (Gupta, Mishra, Bhutani, Hoshing, & Bhalla, 2016). One comparative study assessed the accuracy level between the dental records between private and academic teaching hospitals and showed that students are more likely to be accurate and aware about medicolegal aspects of maintaining dental records rather than dentists in private clinics. In this study, dentists in government sector indeed had better actual knowledge compared with dentists working in private sector.

## 6 LIMITATIONS

The two main types of mass disasters in Saudi Arabia are flash floods during the winter season (victims are usually adventurous hikers) and human stampedes during the annual Hajj pilgrimage. On fewer occasions, cases of dessert outcasts or runaways are reported. The knowledge and skills of dentists in this study might have been limited to victims of these types of disasters, unlike dentists in other countries who were victims of hurricanes, floods, wild fires, earthquakes, volcanoes, and landslides are reported in high rates. Also, the conservative nature of the Saudi community might have made the actual rates of intimate partner abuse and bite‐attacks underreported. Therefore, findings in this study can be generalized to countries with similar weather climates or annual pilgrimage events. This study merely assessed the level of knowledge, without investigating the actual practical skills, such as hands‐on analyses of simulation bite marks and radiographs.

A certain degree of response bias might have occurred when dentists were questioned about their willingness to attend a course, which might have influenced their perceived knowledge. This was controlled by ensuring that the survey was anonymous. Due to the fact that this was a cross‐sectional survey, the relationship between exposures and outcomes is accounted as general association rather than causation. Due to the fact that it was based on a self‐administered survey, a certain degree of recall bias can be present too such as attending course about forensic odontology and analyzing a bite mark in the past. Recruitment of participants was mainly from the capital city of Saudi Arabia, which might be prone to selection bias, but authors do not believe responses would have varied in comparison with dentists in other districts of Saudi Arabia. Despite all these limitations, this study highlights a major gap in the Saudi dental academic curriculum that manifested itself in a poor self‐confidence and a modest level knowledge about forensic odontology among Saudi dentists.

## 7 CONCLUSIONS

The level of knowledge about forensic odontology among dentists in Saudi Arabia was moderate to low. The gap between the perceived and actual knowledge signifies a low self‐confidence in the personal knowledge on this aspect. Although a lack of previous practical experience was present, a strong willingness was observed to pursue further education and training about forensic odontology, especially among younger dentists. Special consideration should be made for less educated (bachelor degree), less experienced (<2 years) dentists, and those working in private clinics as they tend to have poorer knowledge.

## FUNDING INFORMATION

This research did not receive any specific grant from funding agencies in the public, commercial, or not‐for‐profit sectors.
